# Conventionally Fractionated Radiotherapy (CFRT) Versus Stereotactic Body Radiotherapy (SBRT) for Locally Advanced Pancreatic Cancer: A Systematic Review and Meta-Analysis of Comparative Studies

**DOI:** 10.3390/cancers18060971

**Published:** 2026-03-17

**Authors:** Giampaolo Montesi, Marcin Miszczyk, Rita Marina Niespolo, Giorgia Capezzali, Francesco Cellini, Nunziata D’Abbiero, Michele Fiore, Domenico Genovesi, Mariangela La Macchia, Marco Lupattelli, Giovanna Mantello, Fabio Matrone, Luca Nicosia, Nicola Simoni, Pierfrancesco Franco, Francesca De Felice

**Affiliations:** 1 Radiation Oncology Unit, Radiologic Sciences Department, AST Pesaro Urbino, 61121 Pesaro, Italy; 2 Department of Urology, Comprehensive Cancer Center, Medical University of Vienna, 1090 Vienna, Austria; 3 Collegium Medicum, Faculty of Medicine, WSB University, 41-300 Dąbrowa Górnicza, Poland; 4 Radiation Oncology, Fondazione IRCCS San Gerardo dei Tintori, 20900 Monza, Italy; 5 Dipartimento di Diagnostica per Immagini, Radioterapia Oncologica ed Ematologia, Fondazione Policlinico Universitario “A. Gemelli” IRCCS, 00168 Rome, Italy; 6 Dipartimento Universitario Diagnostica per Immagini, Radioterapia Oncologica ed Ematologia, Università Cattolica del Sacro Cuore, 00168 Rome, Italy; 7 Radiotherapy Unit, University Hospital of Parma, 43126 Parma, Italy; 8 Research Unit of Radiation Oncology, Department of Medicine and Surgery, Università Campus Bio-Medico di Roma, 00128 Rome, Italy; 9 Operative Research Unit of Radiation Oncology, Fondazione Policlinico Universitario Campus Bio-Medico di Roma, 00128 Rome, Italy; 10 Department of Radiation Oncology, “S.S. Annunziata” Chieti Hospital, 66100 Chieti, Italy; 11 Department of Medical, Oral and Biotechnological Sciences, “G. D’Annunzio” University of Chieti, 66100 Chieti, Italy; 12 Radiation Oncology Section, Department of Medicine and Surgery, University of Perugia and Perugia General Hospital, 06129 Perugia, Italy; 13 Radiotherapy Department, Azienda Ospedaliero Universitaria delle Marche, 60126 Ancona, Italy; 14 Division of Radiation Oncology, Centro di Riferimento Oncologico di Aviano (CRO), IRCCS, 33081 Aviano, Italy; 15 Advanced Radiation Oncology Department, IRCCS Sacro Cuore Don Calabria Hospital, Cancer Care Center, 37024 Negrar di Valpolicella, Italy; 16 Department of Translational Medicine (DIMET), University of Eastern Piedmont, 28100 Novara, Italy; 17 Department of Radiation Oncology, “Maggiore Della Carità” University Hospital, 28100 Novara, Italy; 18 Radiation Oncology, Policlinico Umberto I, 00161 Rome, Italy; 19 Department of Radiological, Oncological and Pathological Sciences, “Sapienza” University of Rome, 00161 Rome, Italy

**Keywords:** locally advanced pancreatic cancer, pancreatic adenocarcinoma, stereotactic body radiotherapy, chemoradiotherapy, meta-analysis, systematic review

## Abstract

Background: Stereotactic body radiotherapy (SBRT) has gained increasing interest in the treatment of locally advanced pancreatic cancer (LAPC), although its effectiveness has not been defined in randomized trials. This systematic review and meta-analysis aimed to compare clinical outcomes and treatment-related toxicity between SBRT and CFRT in LAPC. Methods: This analysis was performed in accordance with PRISMA guidelines (PROSPERO: CRD420251128943). MEDLINE and Scopus were searched for comparative studies published between January 2015 and July 2025. Five retrospective studies comprising 768 patients fulfilled the eligibility criteria. Pooled hazard ratios (HRs) were calculated for overall survival (OS) and progression-free survival (PFS), while risk ratios (RRs) were estimated for severe (grade ≥ 3) acute toxicity using random-effects models. Study quality was evaluated using the ROBINS-I tool. Results: No significant OS or PFS differences were observed between SBRT and CFRT. SBRT was associated with a lower incidence of severe acute toxicity. The overall risk of bias across studies was moderate. Conclusions: SBRT appears to achieve survival outcomes comparable to CFRT with a favorable acute toxicity profile in patients with LAPC. Nevertheless, the current evidence is limited by retrospective designs and heterogeneity, highlighting the need for prospective randomized trials to define the role of SBRT in this setting.

## Simple Summary

Locally advanced pancreatic cancer (LAPC) represents a major therapeutic challenge, as a large proportion of patients are not candidates for surgical resection. Radiotherapy plays an important role in disease management and may be delivered either through conventionally fractionated radiotherapy (CFRT) over several weeks or through stereotactic body radiotherapy (SBRT) using a small number of high-dose fractions. These approaches differ substantially in treatment schedule, radiation delivery, and toxicity patterns. Because SBRT is increasingly adopted in clinical practice, a clearer understanding of its clinical value compared with CFRT is needed. We therefore reviewed the available comparative studies to better clarify the relative role of CFRT and SBRT in the treatment of patients with LAPC.

## 1. Introduction

Pancreatic cancer accounts for approximately 467,000 deaths annually and continues to rank among the malignancies with the poorest prognosis worldwide, with a 5-year survival rate below 5% [1]. This unfavorable outcome is largely related to its aggressive biological behavior and to the fact that a substantial proportion of patients present with locally advanced or metastatic disease at the time of diagnosis.

As a consequence, curative-intent treatment is feasible only in a limited subset of cases. In particular, patients with locally advanced pancreatic cancer (LAPC) constitute a clinically complex population in whom surgical resection is not an option and treatment strategies are primarily based on systemic therapy, with or without the addition of locoregional approaches.

The role of radiotherapy in LAPC has long been debated. Conventionally fractionated chemoradiotherapy has historically been employed with the intent of enhancing local disease control and, potentially, improving survival. However, the magnitude of its clinical benefit has remained uncertain, in part because of treatment-related toxicity and concerns regarding the interruption or postponement of effective systemic therapy [2].

These unresolved issues have resulted in marked variability in the use of radiotherapy across institutions and treatment eras, reflecting the absence of definitive evidence and uniformly accepted standards. Although stereotactic body radiotherapy (SBRT) has been increasingly introduced into clinical practice for the treatment of LAPC, its effectiveness has not been established through prospective randomized trials [3,4,5,6,7,8]. The available literature is largely derived from retrospective series and prospective non-randomized studies, frequently characterized by heterogeneous patient selection, differences in radiotherapy techniques, and variability in treatment sequencing.

As a result, the comparative value of SBRT relative to conventionally fractionated radiotherapy (CFRT) remains insufficiently defined. At the same time, substantial technical progress has occurred in radiation delivery. Improvements in treatment planning, image guidance, and motion management have enhanced the precision of radiation therapy and reduced uncertainty related to target localization. These developments have facilitated the safe administration of hypofractionated regimens in anatomically challenging sites such as the pancreas. In parallel, the introduction of more effective multi-agent chemotherapy regimens has modified the natural history of LAPC in selected patients, leading to longer disease control and rendering local progression a clinically meaningful event.

In this evolving clinical scenario, the relevance of local tumor control has extended beyond its potential impact on survival [6,7]. Progressive local disease may cause significant morbidity, including pain, biliary obstruction, gastrointestinal complications, and progressive nutritional impairment, all of which can substantially deteriorate quality of life. Consequently, therapeutic strategies that aim to control local disease while maintaining tolerability and allowing uninterrupted systemic therapy have gained increasing attention. SBRT, delivered over a limited number of fractions with high dose per fraction, represents an appealing alternative to prolonged conventionally fractionated schedules. Its shorter overall treatment time may lessen patient burden, reduce the likelihood of treatment-related delays in systemic therapy, and improve logistical feasibility. Nonetheless, uncertainties remain regarding safety, toxicity, and durability of local control, particularly given the close anatomical relationship between pancreatic tumors and surrounding radiosensitive gastrointestinal structures.

Against this background, a direct evaluation of SBRT and CFRT is warranted to better define their respective roles in the management of LAPC. To address this unmet need, we performed a systematic review and meta-analysis comparing SBRT and CFRT in terms of survival outcomes and severe treatment-related toxicity in patients with LAPC, with the objective of providing data that may assist clinical decision-making and guide future research efforts.

## 2. Materials and Methods

This systematic review was conducted in accordance with the PRISMA guidelines. The review protocol was registered in PROSPERO (CRD420251128943).

### 2.1. Search Strategy and Selection Criteria

We searched MEDLINE (via PubMed) and Scopus on 4 August 2025 for studies comparing CFRT with SBRT in the treatment of LAPC. The search was limited to reports published in English between January 2015 and July 2025. Trial registry (clinicaltrials.gov) and abstract books of conferences (European Society of Medical Oncology, American Society of Clinical Oncology and European Society for Radiotherapy and Oncology) were hand searched separately. Backwards citation searching of the reference lists of included reports was performed to identify additional studies of interest. (Supplementary Table S1) Both prospective and retrospective studies were included. Following abstract screening, full-text manuscripts corresponding to the included abstracts were independently screened by two reviewers (GM and FDF). Disagreement was resolved through mediation by a co-author (RMN). In cases where two or more reports were based on overlapping data, only the most recent publication was included in the meta-analysis.

Extracted data were recorded into a standardized database including the following variables: first author’s surname, year of publication, sample size of CFRT group and SBRT group, tumor and treatment details, duration of follow-up, clinical outcomes including hazard ratios (HR) with corresponding confidence intervals (CI) for SBRT compared to CFRT and severe acute toxicity rates. In cases where the HR was not reported in the manuscript, we used Web Plot Digitizer software (https://automeris.io/) v 5.2, to digitize the available graphs and calculate the HRs.

### 2.2. Endpoints

Endpoints. The intent of the analysis was to evaluate overall survival (OS), progression-free survival (PFS), and the proportion of patients experiencing severe (grade ≥ 3) acute toxicity. OS was defined as the time from diagnosis or treatment initiation to death or censoring. PFS was defined as the time from diagnosis or treatment initiation to disease progression, death, or censoring. Survival outcomes were extracted according to the definitions and time origins reported in the original studies.

The number of events of severe acute side effects was extracted from each study when available. To be included in the present analysis, a trial had to report at least one of these three clinical outcomes.

### 2.3. Statistical Analysis

Statistical analysis. Data analysis was performed using Review Manager (RevMan) version 5.4.1. (https://www.cochrane.org) Hazard ratios (HRs) and risk ratios (RRs) were calculated using the random effects Der Simonian-Laird (DL) method. A two-sided *p*-value of <0.05 was considered statistically significant for comparisons. Statistical heterogeneity among studies was assessed using both the Cochrane Q test (with significance set at *p* < 0.1) and the I^2^ value (with I^2^ > 50% indicating substantial heterogeneity) [9].

As our analysis included include non-randomized studies, the Risk Of Bias In Non-randomized Studies—of Interventions (ROBINS-I) tool was used to assess the risk of bias [10].

## 3. Results

**Figure 1:**
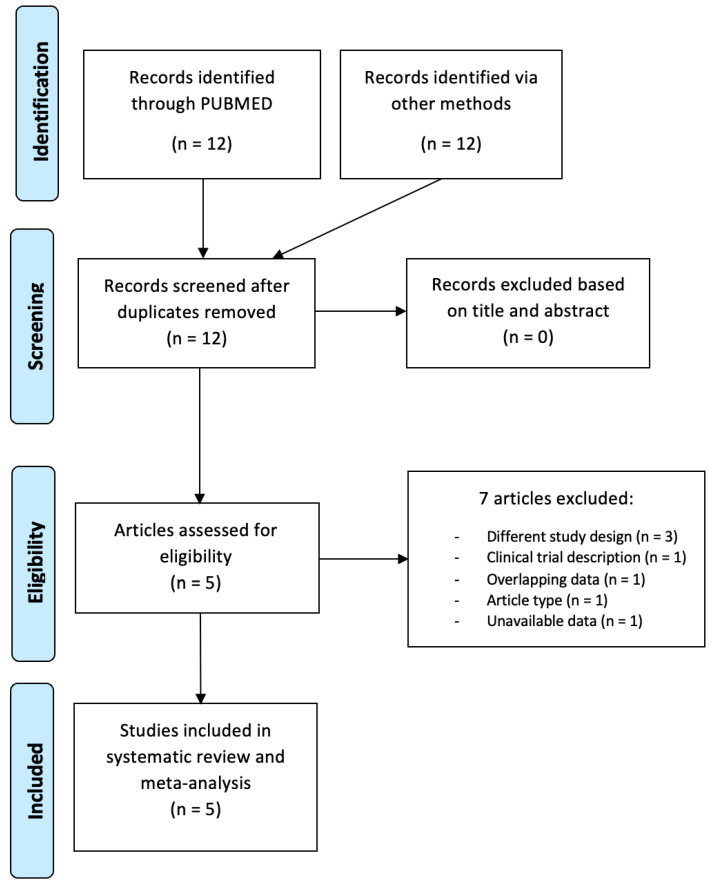
PRISMA flow-chart.

Studies characteristics. Of the 12 records screened, a total of 5 retrospective studies, comprising 768 LAPC patients, were included in qualitative and quantitative analysis [11,12,13,14,15]. Details are showed in Figure 1. The main characteristics of the included studies are presented in Table 1.

**Figure 2:**
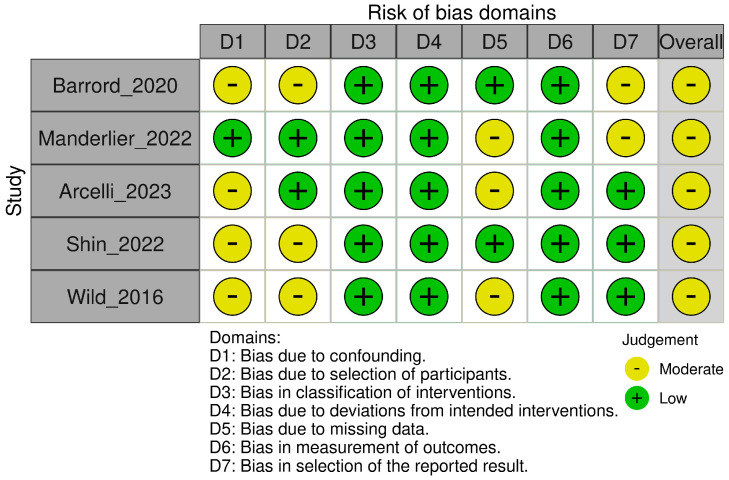
Risk of bias analysis [11,13,14,15,16].

Across all included studies, the overall risk of bias was assessed as moderate, indicating some concerns in study quality but not sufficient to invalidate the results. The main concerns regarded potential confounding and participant selection bias, both of which were judged to be at moderate risk across all included studies. As these were retrospective and non-randomized cohorts, there was limited control over baseline differences between treatment groups, and few studies adequately adjusted for key confounders such as performance status, tumor burden, or receipt of induction chemotherapy. Details are presented in the traffic-light plot (Figure 2). Median follow-up ranged between 15.5 months and 19.7 months in the included studies. Survival outcomes of interest were reported in all but one study. Data on severe acute toxicity were available in two studies, which used different definitions (CTCAE grade ≥ 3 clinical toxicity and severe lymphopenia) [13,14].

Endpoints. Data regarding OS and PFS were available in four studies (*n* = 640) [2,4,7,9] and two studies (*n* = 201) [13,15], respectively.

There was no evidence for significantly different OS in patients treated with SBRT compared to CFRT (HR = 0.74, 95% CI = 0.46–1.20; *p* = 0.22; I^2^ = 76%). The high heterogeneity (I^2^ = 76%) observed is likely due to clinical, methodological and treatment-related differences across studies, including mainly variability in radiotherapy protocols (target delineation, differences in image guidance and motion management) and chemotherapy protocols (type, numbers of cycles and sequence).

Similarly, SBRT was not related with a statistically significant increase in the risk of progression compared to CFRT (HR = 1.89, 95% CI = 0.91–3.95; *p* = 0.09; I^2^ = 61%). While the result approaches statistical significance, it is based on only two studies limiting the strength of evidence. Therefore, the evidence for worse outcomes, does not reach conventional levels of statistical significance.

**Figure 3:**
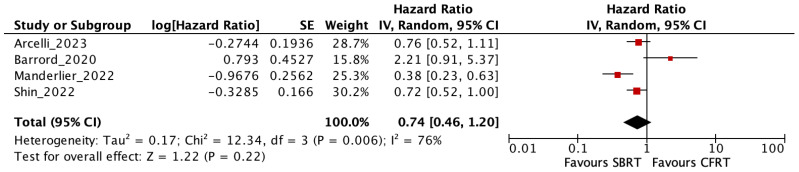
Meta-Analysis of Overall Survival in Patients with Localized Pancreatic Cancer Treated with Stereotactic Body Radiotherapy Versus Conventionally Fractionated Radiotherapy cancer [11,13,15,16].

**Figure 4:**
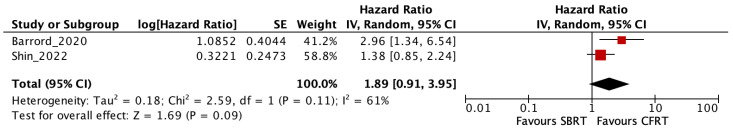
Meta-Analysis of Progression-Free Survival in Patients with Localized Pancreatic Cancer Treated with Stereotactic Body Radiotherapy Versus Conventionally Fractionated Radiotherapy [13,15].

**Figure 5:**
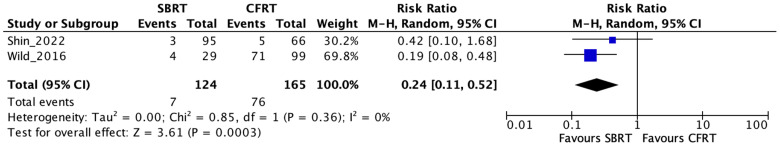
Meta-Analysis of Severe or Worse Acute Toxicity in Patients with Localized Pancreatic Cancer Treated with Stereotactic Body Radiotherapy Versus Conventionally Fractionated Radiotherapy [13,14].

The risk of severe acute toxicity was significantly lower with SBRT (RR = 0.24, 95% CI = 0.11–0.52; *p* < 0.001; I^2^ = 0%). Details are shown in Figure 3, Figure 4 and Figure 5.

## 4. Discussion

The present meta-analysis examines a clinically relevant issue that remains insufficiently resolved in the management of locally advanced pancreatic cancer (LAPC), focusing on the relative contribution of conventionally fractionated radiotherapy (CFRT) and stereotactic body radiotherapy (SBRT). By restricting inclusion to studies directly comparing these two approaches, methodological indirectness is reduced, allowing a more circumscribed interpretation of the available evidence [12]. This aspect is particularly pertinent in a disease context characterized by a limited number of randomized trials and by treatment strategies that have rapidly evolved over time [1,10].

Across the studies analyzed, no statistically significant differences in overall survival (OS) or progression-free survival (PFS) emerged between patients treated with SBRT and those receiving CFRT [12,13,14,15,16]. This observation should not be interpreted as evidence of equivalent oncological efficacy, but rather considered in light of the biological aggressiveness of LAPC. In the majority of patients, prognosis continues to be largely determined by early or occult systemic dissemination, even when local disease control is adequately achieved [11,17,18]. Consequently, intensification of local radiotherapy alone is unlikely to translate into measurable survival benefits unless accompanied by durable systemic disease control.

A numerical, though non-significant, trend toward improved PFS was observed in CFRT cohorts, whereas OS numerically favored SBRT. One possible explanation is the shorter treatment duration of SBRT, which may facilitate earlier resumption or continuation of systemic therapy, a key determinant of survival in locally advanced pancreatic cancer. Conversely, CFRT protocols may involve broader target volumes and regional nodal coverage, potentially contributing to improved locoregional control and a numerical advantage in PFS [2,12,13]. Those finding, however, requires careful interpretation. In routine clinical practice, CFRT is more commonly offered to patients with more favorable baseline characteristics, including preserved performance status, limited comorbidities, and the ability to tolerate prolonged courses of concurrent chemoradiotherapy [11,17]. Conversely, SBRT has often been employed in patients for whom extended treatment schedules are impractical or poorly tolerated, such as those with marginal functional reserve or relevant logistical constraints [13,15]. Although statistical adjustment methods were applied in several studies, residual confounding related to patient selection cannot be excluded and may have contributed to the observed differences [10].

When treatment-related toxicity is considered, a consistent advantage emerges for SBRT. The present analysis shows a significantly lower incidence of severe acute toxicity among patients treated with SBRT compared with those receiving CFRT [12,13,15,16]. This aspect is particularly relevant in LAPC, where treatment feasibility is frequently dictated by tolerability rather than by oncological intent alone. Acute gastrointestinal toxicity and hematologic adverse events associated with concurrent chemoradiotherapy remain important limitations of conventionally fractionated approaches and may lead to treatment interruptions or dose reductions [11,17].

The reduction in severe acute toxicity associated with SBRT is of particular clinical relevance in a patient population often characterized by high symptom burden and limited physiological reserve. Malnutrition, abdominal pain, biliary obstruction, and baseline gastrointestinal dysfunction are common features in LAPC and increase susceptibility to treatment-related adverse effects [11]. In this setting, minimizing acute toxicity is not only relevant for quality of life, but also represents a pragmatic strategy to preserve treatment continuity and maintain eligibility for subsequent systemic therapies [17,18]. However, it should be noted that toxicity definitions differed between studies. In particular, the study by Wild et al. reported severe toxicity based on lymphocyte count reductions, whereas other studies reported CTCAE-defined grade ≥ 3 clinical toxicities [14]. Therefore, this pooled analysis should be interpreted as exploratory.

Differences in treatment logistics further distinguish SBRT from CFRT. While conventionally fractionated regimens typically require daily irradiation over five to six weeks, SBRT is commonly delivered over one to two weeks using a limited number of high-dose fractions [12,18]. This abbreviated treatment course reduces overall treatment time and facilitates earlier initiation or resumption of systemic therapy [11,18]. As reported in previous studies, prolonged delays or interruptions in chemotherapy may adversely affect outcomes, underscoring the importance of maintaining systemic treatment intensity whenever feasible [19,20].

Beyond logistical aspects, SBRT may offer biological advantages related to immune preservation and modulation. Protracted courses of conventionally fractionated radiotherapy expose circulating lymphocytes to repeated low-dose irradiation, frequently resulting in treatment-related lymphopenia [14]. By contrast, SBRT limits radiation exposure to non-target tissues and may exert a relative lymphocyte-sparing effect [14,18]. Given the increasing recognition of immune competence as a determinant of cancer outcomes, this difference may be clinically meaningful, particularly as immunotherapeutic strategies are progressively explored in pancreatic cancer [21,22].

In addition, the high dose per fraction delivered with SBRT has been associated with immunogenic cell death, enhanced antigen release, and modulation of the tumor microenvironment [21,23]. These effects provide a biological rationale for combining SBRT with immune checkpoint inhibitors or other immunomodulatory agents. Early-phase clinical experiences investigating such combinations have reported preliminary signals of activity, although robust evidence in LAPC remains limited and requires further validation [21,23].

Ongoing technical refinements have further improved the safety and applicability of SBRT in pancreatic cancer. Advances in image guidance, respiratory motion management, and target localization have reduced uncertainties related to organ motion and setup variability [14,16]. These developments have enabled dose-escalation strategies in selected patients, allowing delivery of higher biologically effective doses while respecting dose constraints for adjacent organs at risk [16]. Reports describing improved local control with SBRT in carefully selected cohorts support its role as an effective local treatment modality when delivered in experienced centers [7,9].

A potential role for SBRT may also be considered in the setting of borderline resectable disease. Although patients classified as BRPC were included in a subset of the analyzed studies [15,16], data on resection rates and surgical outcomes were not reported in a consistent or systematic manner [13,15,24,25]. Moreover, this population heterogeneity may represent an additional source of variability in survival and toxicity outcomes Against this background, it cannot be excluded that intensified stereotactic strategies—particularly those incorporating dose escalation at the tumour–vessel interface—may facilitate tumor downstaging and enable conversion to surgical resection in selected patients [23,26].

Despite these strengths, several limitations of the available evidence must be acknowledged. All studies included in this meta-analysis were retrospective, exposing the analysis to inherent risks of selection bias, confounding, and heterogeneity in treatment approaches [10]. Data on surgery following SBRT were limited and inconsistently reported across studies, preventing a reliable evaluation of surgical feasibility. Substantial variability was observed across studies in terms of radiotherapy dose and fractionation, target volume definition, the use of concurrent chemotherapy and systemic therapy regimens, as well as outcome assessment, as reflected by the high I^2^ values for pooled survival endpoints [9]. Moreover, toxicity data were reported in only a limited number of studies, limiting the robustness of safety comparisons. In addition, reporting was not fully standardized, and data on late toxicity and quality-of-life outcomes remained limited. Finally, given the small number of included studies and the variability in the time origin used for OS and PFS across studies (e.g., from diagnosis or treatment initiation), publication bias cannot be excluded and the pooled estimates should be interpreted with caution [18,27].

Appropriate patient selection therefore remains a central issue. SBRT may not be suitable for all patients with LAPC, and careful evaluation of tumor extent, proximity to radiosensitive gastrointestinal structures, response to induction chemotherapy, and overall disease biology is required [17,18,19,28,29,30].

## 5. Conclusions

This systematic review and meta-analysis show that SBRT is linked to a reduced severe acute toxicity when compared with CFRT. No statistically significant differences in survival outcomes were identified. Taken together, these considerations contribute to a strengthened clinical rationale for the use of SBRT in routine practice, particularly as a consolidative approach following induction chemotherapy. However, these results should be interpreted with caution given the retrospective design of the included studies and the degree of heterogeneity across the available data. Well-designed prospective randomized trials remain necessary to more clearly define the role of SBRT in the treatment of locally advanced pancreatic cancer.

## Figures and Tables

**Table 1:** Details of the included studies [11,13,14,15,16].

		Patients	Patients	Patients	Patients			
Author	Study	Population	Total	CFRT	SBRT	Median FU m.	Primary Endpoint	Consideration
Barrord_2020	R	BRPC	43	25	18	18.5	LRFS	Only 5 resectable
Manderlier_2022	R	BRPC or LAPC	82	41	41	19.7	median OS	isotoxic dose comparison
Arcelli_2023	R	LAPC cT3–4	354	298	56	16.6	OS, LC, DMFS	excluded CHT-only patients
Shin_2022	R	LAPC	161	66	95	15.5	OS, PFS, FFLP	IC at discretion of physicians
Wild_2016	R	LAPC	128	99	29	12.7	Survival, severe toxicity *	not all patients had data available at each time point

* by lymphocyte counts. Abbreviations: BRPC: borderline resectable pancreatic cancer; CFRT: conventionally fractionated radiotherapy; SBRT: stereotactic body radiotherapy; FU: follow-up; R: retrospective; PC: pancreatic cancer; LAPC: locally advanced pancreatic cancer; LRFS: local-recurrence-free survival; OS: overall survival; LC: local control; DMFS: distant metastasis-free survival; CHT: chemotherapy; PFS: progression-free survival; FFLP: freedom from local progression; IC: induction chemotherapy.

## Data Availability

Data sharing is not applicable to this article as no new data were created or analyzed in this study.

## Supplementary Materials

The following supporting information can be downloaded at: https://www.mdpi.com/article/10.3390/cancers18060971/s1, Table S1. Search strategy.
