# Supplementary material for: Conventionally Fractionated Radiotherapy (CFRT) Versus Stereotactic Body Radiotherapy (SBRT) for Locally Advanced Pancreatic Cancer: A Systematic Review and Meta-Analysis of Comparative Studies
Source: Cancers (Basel). 2026 Mar 17;18(6):971. doi: 10.3390/cancers18060971 (PMC13024495; doi:10.3390/cancers18060971)
Supplement: Supplementary file 1 [file cancers-18-00971-s001.zip › cancers-4160087-supplementary.pdf]

Database / Source	Search strategy	Search date
MEDLINE (PubMed)	(("pancreatic cancer"[Title/Abstract] OR "pancreatic carcinoma"[Title/Abstract]) AND ("locally advanced"[Title/Abstract] OR "borderline resectable"[Title/Abstract]) AND ("chemoradiotherapy"[Title/Abstract] OR "chemoradiation"[Title/Abstract]) AND ("SBRT"[Title/Abstract] OR "stereotactic body radiotherapy"[Title/Abstract] OR "stereotactic ablative radiotherapy"[Title/Abstract]) AND ("comparative study"[Publication Type] OR comparison[Title/Abstract] OR versus[Title/Abstract])) AND ("2015/01/01"[Date - Publication] : "2025/07/31"[Date - Publication])	Jan 2015 – Jul 2025
Scopus	TITLE-ABS-KEY ("pancreatic cancer" OR "pancreatic adenocarcinoma") AND TITLE-ABS-KEY ("SBRT" OR "stereotactic body radiotherapy" OR "stereotactic ablative radiotherapy") AND TITLE-ABS-KEY ("conventional radiotherapy" OR "fractionated radiotherapy" OR "chemoradiotherapy")	Jan 2015 – Jul 2025
ClinicalTrials.gov	Search terms: "pancreatic cancer" AND ("radiotherapy" OR "SBRT"). Filters: interventional studies; completed or recruiting.	Jan 2015 – Jul 2025
Conference abstracts	Hand-search of conference proceedings from major oncology and radiation oncology meetings (ASCO, ASTRO, ESTRO) to identify relevant unpublished studies.	Jan 2015 – Jul 2025
